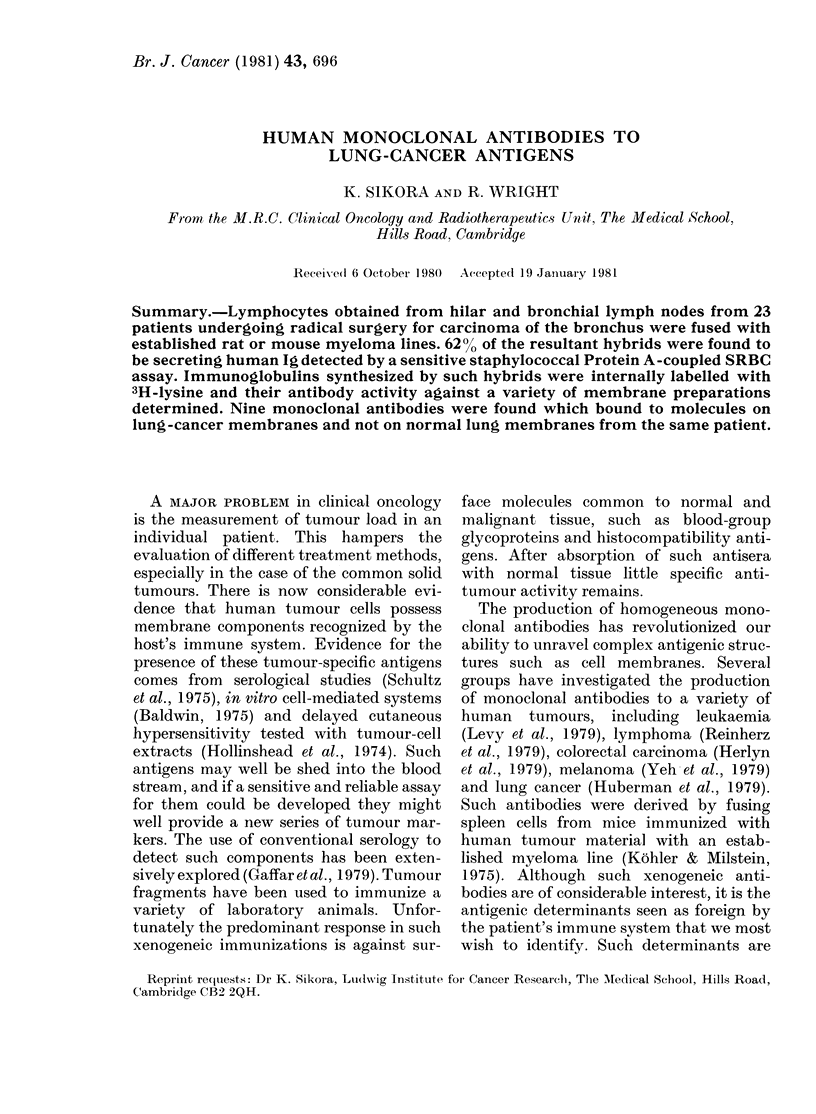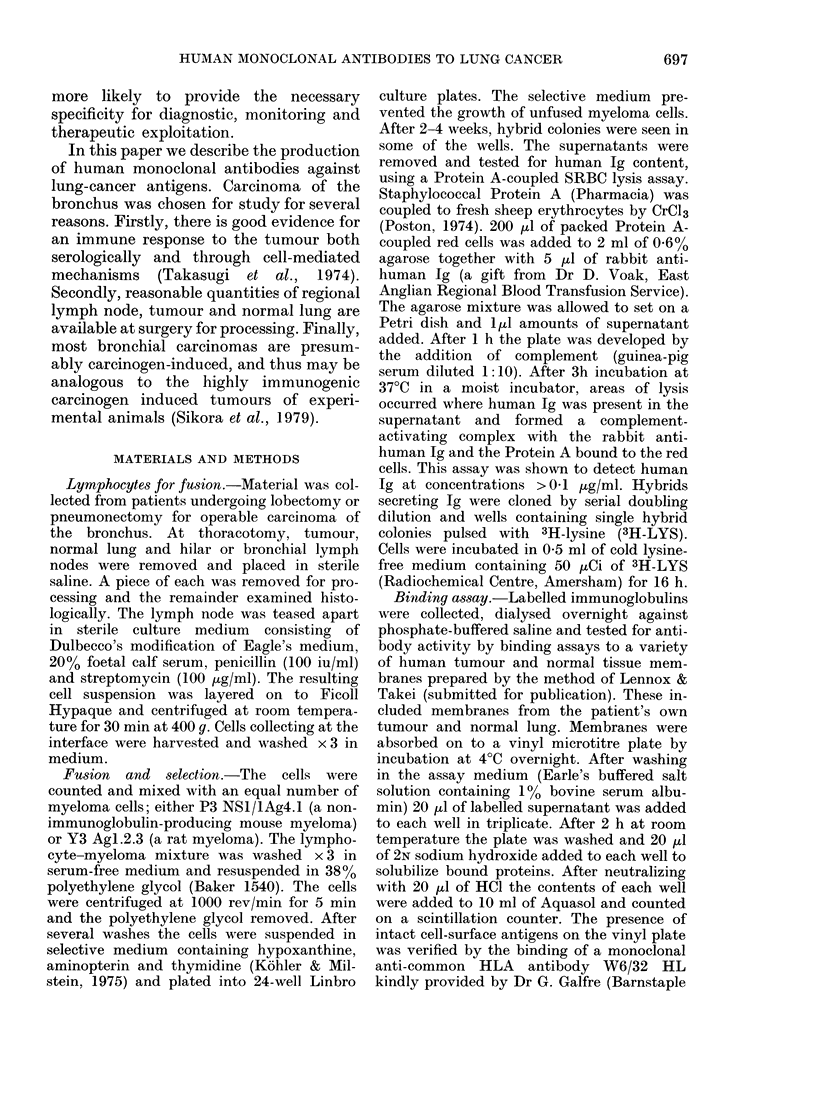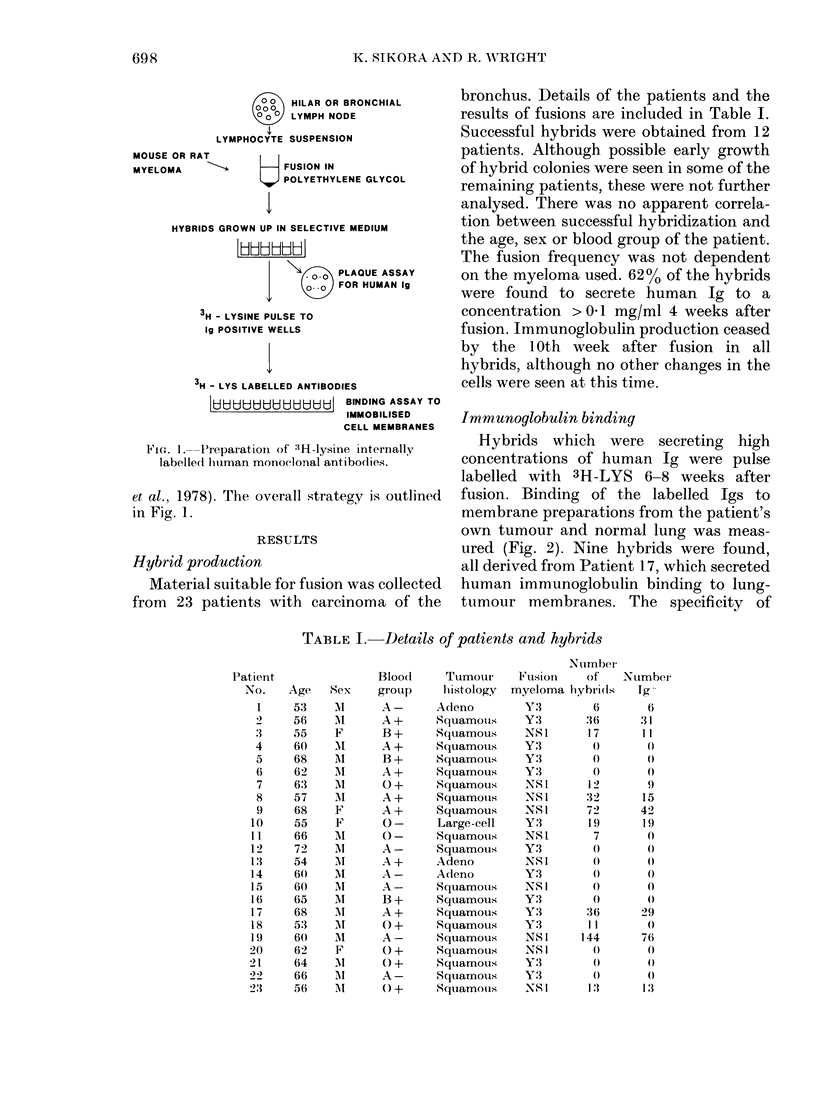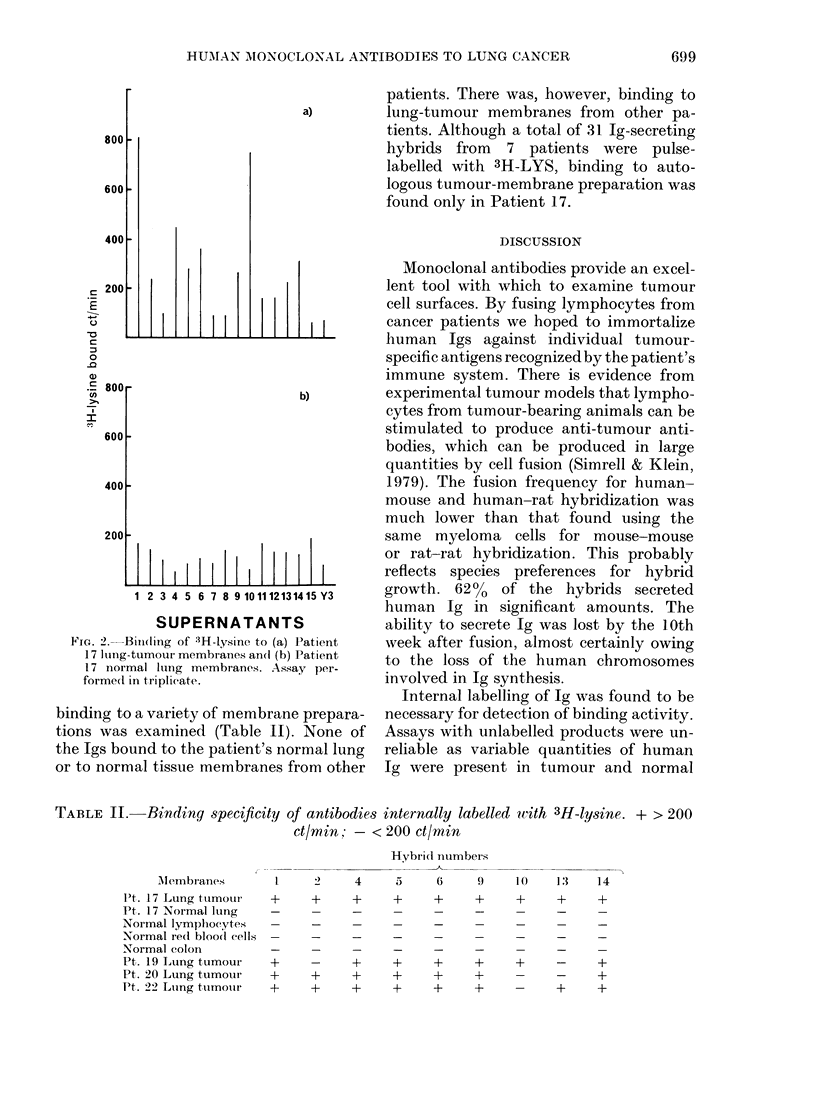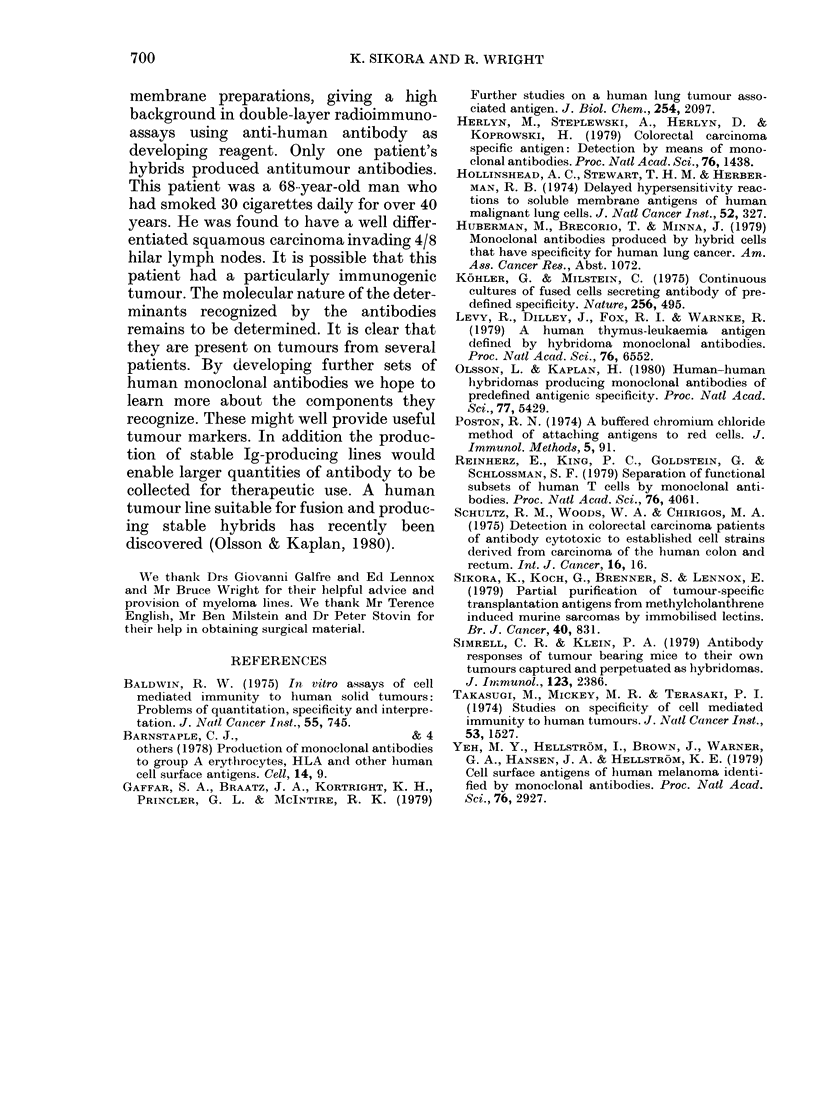# Human monoclonal antibodies to lung-cancer antigens.

**DOI:** 10.1038/bjc.1981.101

**Published:** 1981-05

**Authors:** K. Sikora, R. Wright

## Abstract

Lymphocytes obtained from hilar and bronchial lymph nodes from 23 patients undergoing radical surgery for carcinoma of the bronchus were fused with established rat or mouse myeloma lines. 62% of the resultant hybrids were found to be secreting human Ig detected by a sensitive staphylococcal Protein A-coupled SRBC assay. Immunoglobulins synthesized by such hybrids were internally labelled with 3H-lysine and their antibody activity against a variety of membrane preparations determined. Nine monoclonal antibodies were found which bound to molecules on lung-cancer membranes and not on normal lung membranes from the same patient.


*Br. J. Cancer* (1981) **43**, 696

### HUMAN MONOCLONAL ANTIBODIES TO LUNG-CANCER ANTIGENS

K. SIKORA AND R. WRIGHT

*From the M.R.C. Clinical Oncology and Radiotherapeutics Unit, The Medical School, Hills Road, Cambridge*

Received 6 October 1980 Accepted 19 January 1981

**Summary.—Lymphocytes obtained from hilar and bronchial lymph nodes from 23 patients undergoing radical surgery for carcinoma of the bronchus were fused with established rat or mouse myeloma lines. 62% of the resultant hybrids were found to be secreting human Ig detected by a sensitive staphylococcal Protein A-coupled SRBC assay. Immunoglobulins synthesized by such hybrids were internally labelled with $^3$H-lysine and their antibody activity against a variety of membrane preparations determined. Nine monoclonal antibodies were found which bound to molecules on lung-cancer membranes and not on normal lung membranes from the same patient.**

A MAJOR PROBLEM in clinical oncology is the measurement of tumour load in an individual patient. This hampers the evaluation of different treatment methods, especially in the case of the common solid tumours. There is now considerable evidence that human tumour cells possess membrane components recognized by the host's immune system. Evidence for the presence of these tumour-specific antigens comes from serological studies (Schultz *et al.*, 1975), *in vitro* cell-mediated systems (Baldwin, 1975) and delayed cutaneous hypersensitivity tested with tumour-cell extracts (Hollinshead *et al.*, 1974). Such antigens may well be shed into the blood stream, and if a sensitive and reliable assay for them could be developed they might well provide a new series of tumour markers. The use of conventional serology to detect such components has been extensively explored (Gaffar *et al.*, 1979). Tumour fragments have been used to immunize a variety of laboratory animals. Unfortunately the predominant response in such xenogeneic immunizations is against surface molecules common to normal and malignant tissue, such as blood-group glycoproteins and histocompatibility antigens. After absorption of such antisera with normal tissue little specific anti-tumour activity remains.

The production of homogeneous monoclonal antibodies has revolutionized our ability to unravel complex antigenic structures such as cell membranes. Several groups have investigated the production of monoclonal antibodies to a variety of human tumours, including leukaemia (Levy *et al.*, 1979), lymphoma (Reinherz *et al.*, 1979), colorectal carcinoma (Herlyn *et al.*, 1979), melanoma (Yeh *et al.*, 1979) and lung cancer (Huberman *et al.*, 1979). Such antibodies were derived by fusing spleen cells from mice immunized with human tumour material with an established myeloma line (Köhler & Milstein, 1975). Although such xenogeneic antibodies are of considerable interest, it is the antigenic determinants seen as foreign by the patient's immune system that we most wish to identify. Such determinants are

Reprint requests: Dr K. Sikora, Ludwig Institute for Cancer Research, The Medical School, Hills Road, Cambridge CB2 2QH.

more likely to provide the necessary specificity for diagnostic, monitoring and therapeutic exploitation.

In this paper we describe the production of human monoclonal antibodies against lung-cancer antigens. Carcinoma of the bronchus was chosen for study for several reasons. Firstly, there is good evidence for an immune response to the tumour both serologically and through cell-mediated mechanisms (Takasugi *et al.*, 1974). Secondly, reasonable quantities of regional lymph node, tumour and normal lung are available at surgery for processing. Finally, most bronchial carcinomas are presumably carcinogen-induced, and thus may be analogous to the highly immunogenic carcinogen induced tumours of experimental animals (Sikora *et al.*, 1979).

### MATERIALS AND METHODS

*Lymphocytes for fusion.*—Material was collected from patients undergoing lobectomy or pneumonectomy for operable carcinoma of the bronchus. At thoracotomy, tumour, normal lung and hilar or bronchial lymph nodes were removed and placed in sterile saline. A piece of each was removed for processing and the remainder examined histologically. The lymph node was teased apart in sterile culture medium consisting of Dulbecco's modification of Eagle's medium, 20% foetal calf serum, penicillin (100 iu/ml) and streptomycin (100 $\mu$g/ml). The resulting cell suspension was layered on to Ficoll Hypaque and centrifuged at room temperature for 30 min at 400 *g*. Cells collecting at the interface were harvested and washed $\times 3$ in medium.

*Fusion and selection.*—The cells were counted and mixed with an equal number of myeloma cells; either P3 NS1/1Ag4.1 (a non-immunoglobulin-producing mouse myeloma) or Y3 Ag1.2.3 (a rat myeloma). The lymphocyte–myeloma mixture was washed $\times 3$ in serum-free medium and resuspended in 38% polyethylene glycol (Baker 1540). The cells were centrifuged at 1000 rev/min for 5 min and the polyethylene glycol removed. After several washes the cells were suspended in selective medium containing hypoxanthine, aminopterin and thymidine (Köhler & Milstein, 1975) and plated into 24-well Linbro culture plates. The selective medium prevented the growth of unfused myeloma cells. After 2–4 weeks, hybrid colonies were seen in some of the wells. The supernatants were removed and tested for human Ig content, using a Protein A-coupled SRBC lysis assay. Staphylococcal Protein A (Pharmacia) was coupled to fresh sheep erythrocytes by $CrCl_3$ (Poston, 1974). 200 $\mu$l of packed Protein A-coupled red cells was added to 2 ml of 0·6% agarose together with 5 $\mu$l of rabbit anti-human Ig (a gift from Dr D. Voak, East Anglian Regional Blood Transfusion Service). The agarose mixture was allowed to set on a Petri dish and 1$\mu$l amounts of supernatant added. After 1 h the plate was developed by the addition of complement (guinea-pig serum diluted 1:10). After 3h incubation at 37°C in a moist incubator, areas of lysis occurred where human Ig was present in the supernatant and formed a complement-activating complex with the rabbit anti-human Ig and the Protein A bound to the red cells. This assay was shown to detect human Ig at concentrations $>0{\cdot}1$ $\mu$g/ml. Hybrids secreting Ig were cloned by serial doubling dilution and wells containing single hybrid colonies pulsed with $^3$H-lysine ($^3$H-LYS). Cells were incubated in 0·5 ml of cold lysine-free medium containing 50 $\mu$Ci of $^3$H-LYS (Radiochemical Centre, Amersham) for 16 h.

*Binding assay.*—Labelled immunoglobulins were collected, dialysed overnight against phosphate-buffered saline and tested for antibody activity by binding assays to a variety of human tumour and normal tissue membranes prepared by the method of Lennox & Takei (submitted for publication). These included membranes from the patient's own tumour and normal lung. Membranes were absorbed on to a vinyl microtitre plate by incubation at 4°C overnight. After washing in the assay medium (Earle's buffered salt solution containing 1% bovine serum albumin) 20 $\mu$l of labelled supernatant was added to each well in triplicate. After 2 h at room temperature the plate was washed and 20 $\mu$l of 2N sodium hydroxide added to each well to solubilize bound proteins. After neutralizing with 20 $\mu$l of HCl the contents of each well were added to 10 ml of Aquasol and counted on a scintillation counter. The presence of intact cell-surface antigens on the vinyl plate was verified by the binding of a monoclonal anti-common HLA antibody W6/32 HL kindly provided by Dr G. Galfre (Barnstaple

FIG. 1.—Preparation of $^3$H-lysine internally labelled human monoclonal antibodies.

*et al.*, 1978). The overall strategy is outlined in Fig. 1.

### RESULTS

### *Hybrid production*

Material suitable for fusion was collected from 23 patients with carcinoma of the bronchus. Details of the patients and the results of fusions are included in Table I. Successful hybrids were obtained from 12 patients. Although possible early growth of hybrid colonies were seen in some of the remaining patients, these were not further analysed. There was no apparent correlation between successful hybridization and the age, sex or blood group of the patient. The fusion frequency was not dependent on the myeloma used. 62% of the hybrids were found to secrete human Ig to a concentration $>0{\cdot}1$ mg/ml 4 weeks after fusion. Immunoglobulin production ceased by the 10th week after fusion in all hybrids, although no other changes in the cells were seen at this time.

### *Immunoglobulin binding*

Hybrids which were secreting high concentrations of human Ig were pulse labelled with $^3$H-LYS 6–8 weeks after fusion. Binding of the labelled Igs to membrane preparations from the patient's own tumour and normal lung was measured (Fig. 2). Nine hybrids were found, all derived from Patient 17, which secreted human immunoglobulin binding to lung-tumour membranes. The specificity of

TABLE I.—*Details of patients and hybrids*

| Patient No. | Age | Sex | Blood group | Tumour histology | Fusion myeloma | Number of hybrids | Number Ig$^+$ |
|---|---|---|---|---|---|---|---|
| 1 | 53 | M | A− | Adeno | Y3 | 6 | 6 |
| 2 | 56 | M | A+ | Squamous | Y3 | 36 | 31 |
| 3 | 55 | F | B+ | Squamous | NS1 | 17 | 11 |
| 4 | 60 | M | A+ | Squamous | Y3 | 0 | 0 |
| 5 | 68 | M | B+ | Squamous | Y3 | 0 | 0 |
| 6 | 62 | M | A+ | Squamous | Y3 | 0 | 0 |
| 7 | 63 | M | O+ | Squamous | NS1 | 12 | 9 |
| 8 | 57 | M | A+ | Squamous | NS1 | 32 | 15 |
| 9 | 68 | F | A+ | Squamous | NS1 | 72 | 42 |
| 10 | 55 | F | O− | Large-cell | Y3 | 19 | 19 |
| 11 | 66 | M | O− | Squamous | NS1 | 7 | 0 |
| 12 | 72 | M | A− | Squamous | Y3 | 0 | 0 |
| 13 | 54 | M | A+ | Adeno | NS1 | 0 | 0 |
| 14 | 60 | M | A− | Adeno | Y3 | 0 | 0 |
| 15 | 60 | M | A− | Squamous | NS1 | 0 | 0 |
| 16 | 65 | M | B+ | Squamous | Y3 | 0 | 0 |
| 17 | 68 | M | A+ | Squamous | Y3 | 36 | 29 |
| 18 | 53 | M | O+ | Squamous | Y3 | 11 | 0 |
| 19 | 60 | M | A− | Squamous | NS1 | 144 | 76 |
| 20 | 62 | F | O+ | Squamous | NS1 | 0 | 0 |
| 21 | 64 | M | O+ | Squamous | Y3 | 0 | 0 |
| 22 | 66 | M | A− | Squamous | Y3 | 0 | 0 |
| 23 | 56 | M | O+ | Squamous | NS1 | 13 | 13 |

FIG. 2.—Binding of $^3$H-lysine to (a) Patient 17 lung-tumour membranes and (b) Patient 17 normal lung membranes. Assay performed in triplicate.

binding to a variety of membrane preparations was examined (Table II). None of the Igs bound to the patient's normal lung or to normal tissue membranes from other patients. There was, however, binding to lung-tumour membranes from other patients. Although a total of 31 Ig-secreting hybrids from 7 patients were pulse-labelled with $^3$H-LYS, binding to autologous tumour-membrane preparation was found only in Patient 17.

### DISCUSSION

Monoclonal antibodies provide an excellent tool with which to examine tumour cell surfaces. By fusing lymphocytes from cancer patients we hoped to immortalize human Igs against individual tumour-specific antigens recognized by the patient's immune system. There is evidence from experimental tumour models that lymphocytes from tumour-bearing animals can be stimulated to produce anti-tumour antibodies, which can be produced in large quantities by cell fusion (Simrell & Klein, 1979). The fusion frequency for human–mouse and human–rat hybridization was much lower than that found using the same myeloma cells for mouse–mouse or rat–rat hybridization. This probably reflects species preferences for hybrid growth. 62% of the hybrids secreted human Ig in significant amounts. The ability to secrete Ig was lost by the 10th week after fusion, almost certainly owing to the loss of the human chromosomes involved in Ig synthesis.

Internal labelling of Ig was found to be necessary for detection of binding activity. Assays with unlabelled products were unreliable as variable quantities of human Ig were present in tumour and normal

TABLE II.—*Binding specificity of antibodies internally labelled with $^3$H-lysine.* $+ > 200$ *ct/min;* $- < 200$ *ct/min*

| | Hybrid numbers | | | | | | | | |
|---|---|---|---|---|---|---|---|---|---|
| Membranes | 1 | 2 | 4 | 5 | 6 | 9 | 10 | 13 | 14 |
| Pt. 17 Lung tumour | + | + | + | + | + | + | + | + | + |
| Pt. 17 Normal lung | – | – | – | – | – | – | – | – | – |
| Normal lymphocytes | – | – | – | – | – | – | – | – | – |
| Normal red blood cells | – | – | – | – | – | – | – | – | – |
| Normal colon | – | – | – | – | – | – | – | – | – |
| Pt. 19 Lung tumour | + | – | + | + | + | + | + | – | + |
| Pt. 20 Lung tumour | + | + | + | + | + | + | – | – | + |
| Pt. 22 Lung tumour | + | + | + | + | + | + | – | + | + |

membrane preparations, giving a high background in double-layer radioimmunoassays using anti-human antibody as developing reagent. Only one patient's hybrids produced antitumour antibodies. This patient was a 68-year-old man who had smoked 30 cigarettes daily for over 40 years. He was found to have a well differentiated squamous carcinoma invading 4/8 hilar lymph nodes. It is possible that this patient had a particularly immunogenic tumour. The molecular nature of the determinants recognized by the antibodies remains to be determined. It is clear that they are present on tumours from several patients. By developing further sets of human monoclonal antibodies we hope to learn more about the components they recognize. These might well provide useful tumour markers. In addition the production of stable Ig-producing lines would enable larger quantities of antibody to be collected for therapeutic use. A human tumour line suitable for fusion and producing stable hybrids has recently been discovered (Olsson & Kaplan, 1980).

We thank Drs Giovanni Galfre and Ed Lennox and Mr Bruce Wright for their helpful advice and provision of myeloma lines. We thank Mr Terence English, Mr Ben Milstein and Dr Peter Stovin for their help in obtaining surgical material.

### REFERENCES

BALDWIN, R. W. (1975) *In vitro* assays of cell mediated immunity to human solid tumours: Problems of quantitation, specificity and interpretation. *J. Natl Cancer Inst.*, **55**, 745.

BARNSTAPLE, C. J., & 4 others (1978) Production of monoclonal antibodies to group A erythrocytes, HLA and other human cell surface antigens. *Cell*, **14**, 9.

GAFFAR, S. A., BRAATZ, J. A., KORTRIGHT, K. H., PRINCLER, G. L. & MCINTIRE, R. K. (1979) Further studies on a human lung tumour associated antigen. *J. Biol. Chem.*, **254**, 2097.

HERLYN, M., STEPLEWSKI, A., HERLYN, D. & KOPROWSKI, H. (1979) Colorectal carcinoma specific antigen: Detection by means of monoclonal antibodies. *Proc. Natl Acad. Sci.*, **76**, 1438.

HOLLINSHEAD, A. C., STEWART, T. H. M. & HERBERMAN, R. B. (1974) Delayed hypersensitivity reactions to soluble membrane antigens of human malignant lung cells. *J. Natl Cancer Inst.*, **52**, 327.

HUBERMAN, M., BRECORIO, T. & MINNA, J. (1979) Monoclonal antibodies produced by hybrid cells that have specificity for human lung cancer. *Am. Ass. Cancer Res.*, Abst. 1072.

KÖHLER, G. & MILSTEIN, C. (1975) Continuous cultures of fused cells secreting antibody of predefined specificity. *Nature*, **256**, 495.

LEVY, R., DILLEY, J., FOX, R. I. & WARNKE, R. (1979) A human thymus-leukaemia antigen defined by hybridoma monoclonal antibodies. *Proc. Natl Acad. Sci.*, **76**, 6552.

OLSSON, L. & KAPLAN, H. (1980) Human–human hybridomas producing monoclonal antibodies of predefined antigenic specificity. *Proc. Natl Acad. Sci.*, **77**, 5429.

POSTON, R. N. (1974) A buffered chromium chloride method of attaching antigens to red cells. *J. Immunol. Methods*, **5**, 91.

REINHERZ, E., KING, P. C., GOLDSTEIN, G. & SCHLOSSMAN, S. F. (1979) Separation of functional subsets of human T cells by monoclonal antibodies. *Proc. Natl Acad. Sci.*, **76**, 4061.

SCHULTZ, R. M., WOODS, W. A. & CHIRIGOS, M. A. (1975) Detection in colorectal carcinoma patients of antibody cytotoxic to established cell strains derived from carcinoma of the human colon and rectum. *Int. J. Cancer*, **16**, 16.

SIKORA, K., KOCH, G., BRENNER, S. & LENNOX, E. (1979) Partial purification of tumour-specific transplantation antigens from methylcholanthrene induced murine sarcomas by immobilised lectins. *Br. J. Cancer*, **40**, 831.

SIMRELL, C. R. & KLEIN, P. A. (1979) Antibody responses of tumour bearing mice to their own tumours captured and perpetuated as hybridomas. *J. Immunol.*, **123**, 2386.

TAKASUGI, M., MICKEY, M. R. & TERASAKI, P. I. (1974) Studies on specificity of cell mediated immunity to human tumours. *J. Natl Cancer Inst.*, **53**, 1527.

YEH, M. Y., HELLSTRÖM, I., BROWN, J., WARNER, G. A., HANSEN, J. A. & HELLSTRÖM, K. E. (1979) Cell surface antigens of human melanoma identified by monoclonal antibodies. *Proc. Natl Acad. Sci.*, **76**, 2927.